# An Integrated Academic Oncology Ecosystem for Hawaiʻi and the U.S.-Affiliated Pacific Islands

**DOI:** 10.3390/cancers18091441

**Published:** 2026-04-30

**Authors:** Stephanie J. Si Lim, Hideko Yamauchi, Teruo Yamauchi, Kenneth Sumida, John Shepherd, Thomas Samuel Shomaker, Lee E. Buenconsejo-Lum, Naoto T. Ueno

**Affiliations:** 1 University of Hawaiʻi Cancer Center, Honolulu, HI 96813, USA; ssi@cc.hawaii.edu (S.J.S.L.); hyamauchi@cc.hawaii.edu (H.Y.); tyamauchi@cc.hawaii.edu (T.Y.); knsumida@hawaii.edu (K.S.); jshepherd@cc.hawaii.edu (J.S.); lbuencon@hawaii.edu (L.E.B.-L.); 2 John A. Burns School of Medicine, Honolulu, HI 96813, USA; shomaker@hawaii.edu

**Keywords:** cancer disparities, Hawaiʻi, U.S.-Affiliated Pacific Islands, oncology workforce, telehealth, clinical trials, implementation cancer disparities, Hawaiʻi, U.S.-Affiliated Pacific Islands, oncology workforce, telehealth, clinical trials, implementation

## Abstract

**Background**: Delivering comprehensive cancer prevention, diagnosis, and treatment across Hawaiʻi and the U.S.-Affiliated Pacific Islands (USAPI) is constrained by geographic isolation, oncology workforce shortages, and persistent cancer inequities. **Objectives**: The University of Hawaiʻi Cancer Center, the state’s only National Cancer Institute-designated cancer center, partners with community healthcare systems to address cancer health disparities. Here, we describe an implementation-focused strategy initiated in December 2024 that is designed to improve equitable access to evidence-based oncology services across the catchment area. **Approach**: This program description integrates publicly available demographic and health system data and presents a structured implementation framework centered on (1) workforce development and oncology training pathways; (2) a statewide clinical oncology network supported by telehealth; (3) community-engaged screening and early detection outreach; and (4) strengthening clinical research and trial infrastructure with deliberate inclusion of underserved populations. **Evaluation**: We outline an evaluation framework incorporating process and outcome metrics spanning workforce capacity, screening participation, timeliness of care, clinical trial enrollment, and equity indicators stratified by county, island, and population group. **Conclusions**: This approach offers a scalable, implementation-oriented model for developing an academic oncology ecosystem that emphasizes measurement, accountability, and equity, with potential applicability to other geographically dispersed and ethnically diverse regions.

## Simple Summary

Ka ʻUmeke Lama is a new, early-phase initiative established by the University of Hawaiʻi Cancer Center and John A. Burns School of Medicine to address cancer inequities across Hawai’i and the U.S.-Affiliated Pacific Islands (USAPI) by building an integrated academic oncology ecosystem grounded in implementation science principles. This initiative is organized around three mutually reinforcing pillars—workforce education and development, a telehealth-enabled clinical network with community partnerships, and a hub-and-spoke clinical research infrastructure—designed to extend oncology expertise and clinical trial access beyond East O’ahu to neighbor islands and the USAPI in a culturally responsive way. This perspective describes the regional context and tiered disparity landscape and outlines how Ka ʻUmeke Lama is structured to influence reach, adoption, fidelity, and sustainability of evidence-informed cancer care, and proposes a pragmatic evaluation framework with equity-focused metrics that can be phased in as data systems mature.

## 1. Introduction

Hawaiʻi is geographically isolated from major U.S. academic medical centers, and oncology services are distributed across multiple islands. County-level population estimates indicate that approximately 69% of residents live in Honolulu County, with the remaining ~31% residing on the neighbor islands (Maui, Hawaiʻi Island, Kauaʻi, Molokaʻi, Lānaʻi) [1]. This equates to a total of 1.4 million people living in the State of Hawaiʻi [1,2]. These geographic realities amplify access barriers for communities requiring specialized cancer services, particularly when care requires frequent visits, multidisciplinary therapies, and closer monitoring of treatment-related toxicities [3]. Even relatively short inter-island flights impose costs, logistical complexity, and psychosocial burden, especially for patients who are older, medically fragile, or socioeconomically disadvantaged.

The USAPI comprises three U.S. territories (American Samoa, Guam, and the Commonwealth of the Northern Mariana Islands) and three Freely Associated States (the Federated States of Micronesia, the Republic of the Marshall Islands, and the Republic of Palau) [4]. Together, these jurisdictions include hundreds of remote islands and atolls and are home to more than 440,000 people [4,5]. As the only NCI-designated cancer center serving the State of Hawaiʻi, the University of Hawai’i Cancer Center (UH Cancer Center) catchment area includes Hawaiʻi and the USAPI, creating an obligation to pursue cancer control strategies that are both clinically robust and locally feasible [6].

Across Hawaiʻi and the USAPI, cancer inequities reflect intersecting social drivers of health, constrained health system capacity, and structural barriers to prevention, early detection, and treatment [7,8,9]. For example, recent USAPI registry analyses show lower overall breast cancer incidence than the continental U.S., but substantially higher proportions of late-stage diagnoses, underscoring gaps in screening, timely diagnosis, and access to treatment [10]. Similarly, cervical cancer incidence in parts of the USAPI remains markedly elevated, highlighting the urgency of culturally grounded prevention and screening efforts [11]. Traditional models of cancer care delivery, which assume reasonable proximity to tertiary centers and relatively dense specialty workforce, are not well adapted to communities with unique geographic, racial, and socioeconomic differences. Fragmented outreach clinics and ad hoc telehealth arrangements may offer short-term relief, but they rarely generates sustainable, system-level change. As such, there is a clear need for an integrated, implementation-focused academic oncology ecosystem that links academic expertise with community-based care delivery across Hawai’i and the USAPI. Herein, we describe Ka ʻUmeke Lama, a multi-pillar initiative in the early implementation phase that is aimed at building such an ecosystem through coordinated efforts in workforce development, clinical network infrastructure, clinical research, and community partnership. We outline the regional context and tiers of cancer health disparities, detail the program’s design, and propose an evaluation framework guided by principles such as phased implementation, iterative adaptation, and continuous feedback using routine data to foster learning and adaptation. This implementation framework is conceptually aligned with established models such as RE-AIM (Reach, Effectiveness, Adoption, Implementation, and Maintenance) and learning health system principles, which emphasize iterative evaluation, real-world data integration, and continuous system adaptation.

## 2. Health Disparity Categories in Hawaiʻi and the U.S.-Affiliated Pacific Islands

Health disparities across the UH Cancer Center catchment area exist along a continuum rather than as a single, uniform challenge. Within Hawai’i, differences in geographic access, socioeconomic conditions, and health system infrastructure create distinct tiers of cancer care access and outcomes, which are further magnified when the USAPI are considered. As the only NCI-designated cancer center in the Pacific, it is of utmost importance that the UH Cancer Center recognizes these barriers in order to design interventions that are both equitable and pragmatic, allowing resources to be aligned with need and implementation strategies to be tailored to local realities.

For example, within Oʻahu (population ~1 million), meaningful differences are evident: East Oʻahu benefits from proximity to tertiary and academic cancer care, while non-East Oʻahu communities experience greater barriers related to transportation, care coordination, and timely access to specialty services. Physicians provide care through existing community healthcare systems. The UH Cancer Center does not have its own cancer care service but historically has provided clinical trial support, which has been most accessible to the East Oahu physicians due to physical proximity, health system support, and physician engagement. These disparities are more pronounced on the neighbor islands and in the USAPI, where limited oncology workforce capacity and the need for inter-island travel contribute to delays in diagnosis, treatment, and access to clinical trials. The most profound disparities are seen in the USAPI, where vast geographic distances, constrained infrastructure, workforce shortages, and cultural and language differences create systemic barriers to cancer prevention, early detection, and treatment.

Table 1 summarizes the four tiers of relative disparity severity across the UH Cancer Center catchment area, ranging from lowest (East O’ahu) to highest (USAPI). This tiered framework is not meant to oversimplify complex realities but rather to guide strategic planning. In East O’ahu, the primary focus is on optimizing quality, ensuring that patients benefit from cutting-edge therapies, precision oncology, and robust survivorship services. In non-East O’ahu communities, strategies emphasize care coordination, navigation, and decentralized screening and trials to mitigate barriers related to transportation and system navigation. On the neighbor islands, tele-oncology, hub-and-spoke models, mobile screening, and regional trial activation become central tools for increasing access and reducing the need for inter-island travel. In the USAPI, the emphasis shifts toward capacity building, regional partnerships, prevention and early detection, and global oncology collaborations that leverage shared resources and remote expertise.

### Regional Context and Drivers of Inequity

Geographic cancer health disparities in Hawaiʻi and the USAPI are influenced by interrelated contextual factors that shape both individual-level behavior and system-level capacity. Key drivers include:

1. Transportation barriers resulting in geographic isolation and inter-island or inter-atoll travel requirements, which increase the time and cost of accessing care and may delay or prevent completion of recommended treatment.
2. Shortages of oncology-specialized healthcare providers, including physicians, nurses, advanced practice providers, and allied health professionals, which may limit local capacity to deliver guideline-concordant care.
3. Limited subspecialty care outside Honolulu, particularly for radiation oncology, subspecialty surgery, and complex systemic therapy, leading to dependence on referrals and travel.
4. Restricted health funding and lower per capita health expenditure in many Pacific jurisdictions compared with the continental U.S.A., constraining investment in infrastructure, diagnostics, and therapeutics.
5. Increasing cancer burden amid population aging, with older adults accounting for a growing proportion of cancer incidence and survivors, placing pressure on already strained systems.
6. Lower acceptance and utilization of established health and cancer screening in some communities, driven by historical trauma, mistrust, cultural beliefs, language barriers, and competing socioeconomic priorities.

Addressing these drivers requires approaches that move beyond traditional clinic-centric models and that actively incorporate community voices, cultural frameworks, and cross-sector partnerships. Ka ʻUmeke Lama explicitly builds on this recognition, using the disparity tiers and contextual drivers to prioritize interventions and sequence implementation activities across the catchment area.

## 3. Oncology Workforce Constraints

Statewide workforce assessment reports estimate an overall physician shortfall of about 21%, reflecting the gap between available full-time equivalents and modeled demand for services [12,13]. Neighbor-island counties face more severe shortages, with prior analyses suggesting deficits of roughly 30–40% or higher compared with Oʻahu [12,14]. Within this broader shortage, cancer care is particularly affected: oncology has been identified as one of the subspecialties with the greatest unmet need in Hawaiʻi, with some reports describing the near-absence of oncology services in certain neighbor-island counties and very high estimated shortfall percentages [13,15,16]. These deficits extend beyond physicians to include oncology nurses, advanced practice providers, navigators, social workers, and other key members of the multidisciplinary cancer care team [15,16].

Residents of Maui, Hawaiʻi Island, Kauaʻi, and rural communities across the state frequently must travel by air to access specialty cancer care, compounding delays in diagnosis and treatment [14,17]. Focus group work in rural Hawaiʻi has documented how limited local oncology capacity, fragmented coordination, and reliance on fly-in providers hinder the delivery of guideline-concordant care and timely follow-up [17]. Patients may receive portions of their care across multiple health systems or islands, with limited interoperability of records and variable access to tumor boards or molecular testing. These structural challenges increase the risk of treatment discontinuity and exacerbate the burden on patients and families who must act as their own care coordinators.

These access barriers intersect with long-standing cancer disparities affecting Native Hawaiian, Pacific Islander, and Filipino communities, who already experience higher incidence and mortality from several major cancers [18,19,20]. New statewide initiatives in cancer care and workforce development explicitly frame the oncology workforce crisis—especially on neighbor islands and in the USAPI—as a barrier to equitable, evidence-based treatment that needs to be addressed urgently [16,21,22]. Importantly, oncology workforce constraints in Hawai’i and the Pacific reflect national and global trends in rural and underserved settings, suggesting that solutions developed here may have broader relevance.

### 3.1. Population Aging and Cancer Demand

Hawaiʻi is experiencing rapid population aging. State planning offices project that residents aged 65 and older will comprise more than one-fifth of the state’s population by 2030 and continue to rise thereafter [23,24,25]. The Hawaiʻi State Department of Health has projected an increase of nearly 90% in the number of residents aged 65 and older between 2018 and 2040 [23,26,27]. Because cancer incidence and prevalence increase sharply with age, national projections anticipate that older adults will account for a growing majority of cancer survivors, with models suggesting that approximately 70% or more of survivors may be 65 or older by 2040 [28]. For Hawaiʻi, this “silver tsunami” is expected to translate into rising demand not only for complex cancer treatment but also for geriatric assessment, management of multimorbidity and polypharmacy, survivorship planning, and supportive and palliative care tailored to our kūpuna, or elderly individuals [15,28].

### 3.2. Implications for Evidence-Based Cancer Care

Against this backdrop of high need and limited workforce, workforce shortages pose an immediate barrier to the implementation of contemporary, evidence-based oncology pathways across the state [21,29,30]. Insufficient numbers of oncology specialists and support staff constrain capacity for timely consultation, evaluation, and molecular testing, multidisciplinary tumor boards, clinical trial participation, and the delivery of complex systemic therapies and radiation regimens [21,29,30].

For older adults, limited availability of geriatric-trained clinicians and supportive care professionals makes it difficult to systematically perform geriatric assessments, optimize function, and integrate patient goals and social determinants into cancer care plans [15,31]. This can lead to fragmented or reactive care rather than proactive, patient-centered planning. In the USAPI, constrained resources further limit oncology capacity, with per capita health expenditures in several jurisdictions far below U.S. levels, reinforcing the need for cost-effective shared infrastructure, telehealth, and regional referral pathways that reduce the need for travel while supporting quality and safety.

Addressing these converging trends will require coordinated investment in workforce training, recruitment and retention, particularly on neighbor islands, as well as novel programs such as tele-oncology and team-based models, and community-engaged strategies to ensure that all patients in Hawaiʻi can access guideline-concordant, culturally grounded cancer care [21,29,30,31].

## 4. Integrated Academic Oncology Ecosystem: Ka ʻUmeke Lama

To address these challenges described above, we established an integrated academic oncology ecosystem tailored to Hawaiʻi and the USAPI in December 2024 through a partnership between the UH Cancer Center and the John A. Burns School of Medicine (JABSOM). The core premise is that durable improvements in cancer outcomes and equity require coordinated, long-term investment across four areas: workforce development, clinical network infrastructure, research capacity, including clinical trials, and community partnership. Within an implementation science lens, Ka ʻUmeke Lama is designed to influence key implementation dimensions—reach, adoption, fidelity, and sustainability—rather than to function as a single, time-limited intervention.

We named this initiative Ka ʻUmeke Lama, reflecting Hawaiʻi’s cultural context and our commitment to a respectful, community-engaged approach. The name conveys the image of a bowl of knowledge that guides healing—symbolizing shared wisdom, collaboration, and responsibility. From its inception, the program emphasized implementation-focused components and measurable objectives to support academic evaluation, accountability, and potential replication in other regions. The ecosystem is explicitly structured to expand the reach of oncology expertise beyond East O’ahu, support adoption of evidence-informed care models by community sites, promote fidelity to agreed-upon pathways and protocols, and build the organizational and financial conditions needed for sustainability over time.

The initiative is organized around three key pillars that operationalize the broader ecosystem concept: [1] strengthening clinical research and access to innovation, [2] advancing statewide professional education and workforce development, and [3] addressing health disparities across the neighbor islands and USAPI through clinical networks and community partnerships. These pillars are mutually reinforcing. Education and workforce development influence adoption and fidelity by equipping teams with shared competencies and mental models. Clinical research infrastructure supports the reach of innovation to underserved communities and creates incentives for sustained academic–community collaboration. Clinical networks and community partnerships extend the reach of services and help ensure that implementation strategies are acceptable, culturally grounded, and embedded in local systems in ways that can be maintained. Finally, this implementation framework is conceptually aligned with established models such as RE-AIM (Reach, Effectiveness, Adoption, Implementation, and Maintenance) and learning health system principles, which emphasize iterative evaluation and continuous system adaptation.

### 4.1. Education and Workforce Development

The first pillar—statewide professional education and workforce development—focuses on building and maintaining a skilled oncology workforce capable of delivering high-quality, team-based care across diverse, geographically dispersed settings. The initiative creates oncology-focused training pathways that span the continuum from undergraduate and graduate medical education to continuing professional development, including a medical oncology fellowship rotation structure that incorporates experiences on neighbor islands and telehealth-supported community practices. These pathways are explicitly intended to increase the reach of specialized oncology training to clinicians who practice outside Honolulu and to support the adoption of standardized, evidence-based care processes across multiple sites. The first medical oncology fellowship class will start in July 2027.

Beyond physician training, Ka ʻUmeke Lama emphasizes the critical roles of advanced practice providers, oncology pharmacists, genetic counselors, nutritionists, oncology social workers, and navigators in delivering comprehensive care. Educational activities include case-based tele-monitoring, shared clinical protocols, and multidisciplinary virtual tumor boards that connect providers across islands and, where feasible, with USAPI partners. In fact, the early establishment of a multidisciplinary virtual sarcoma and breast cancer tumor board at the UH Cancer Center has been implemented with early engagement across sites; its impact will be formally evaluated in future analyses. By using common curricula and shared clinical pathways, the program seeks to enhance fidelity—that is, the degree to which care delivered in neighbor-island and USAPI settings aligns with agreed-upon standards and guidelines—while allowing for culturally responsive local adaptation. Longitudinal mentorship, career development opportunities, and alignment with institutional workforce strategies are intended to support the sustainability of this pillar, both in terms of personnel retention and ongoing program delivery.

### 4.2. Clinical Research and Access to Innovation

The second pillar aims to strengthen research and clinical trial infrastructure to reduce disparities in access to emerging cancer therapies. Historically, most clinical trials in Hawai’i have been concentrated in East O’ahu, limiting participation among patients on neighbor islands and in the USAPI. By establishing standardized processes for regulatory navigation, biospecimen handling, and trial activation across partner sites, the program aims to expand equitable access to clinical trials beyond East Oʻahu.

At the hub, the UH Cancer Center provides centralized Institutional Review Board (IRB) reliance, protocol development and management, regulatory oversight, and data coordination. Satellite sites engage at varying levels depending on their readiness and resources. Some will serve as primarily referring sites, where potentially eligible patients are identified and connected to hub-based trials through telehealth consultation and navigation. Sites with greater infrastructure and staffing will participate directly as trial satellite sites, with capacity to perform specified clinical and research procedures under the umbrella of UH Cancer Center oversight. This structure is intended to reduce duplicative regulatory burden, support consistent trial conduct across sites, and make research participation more accessible to patients who live far from Oahu.

Early implementation will intentionally focus on trials that are logically feasible for satellite participation. Priority will be given to studies that use oral agents and that require a relatively small number of on-site procedures that can be delivered safely in community settings. Trials with highly specialized procedures or intensive monitoring requirements will initially remain hub-based, with patients referred from satellite sites when appropriate. In addition, Ka ʻUmeke Lama will explore remote pre-screening and consent models, including telehealth-based eligibility assessment and electronic or remote consent processes where allowed by regulation and institutional policy. These approaches are designed to reduce the need for travel solely for trial evaluation and to embed research discussions earlier in patients’ care journeys.

Biospecimen handling is another critical component of the research pillar. The program will rely on standardized operating procedures developed and maintained by the UH Cancer Center for specimen collection, processing, labeling, storage, and shipment. A centralized biorepository at the UH Cancer Center will coordinate long-term storage and distribution of biospecimens, ensuring uniform quality control and adherence to regulatory and ethical standards. Predefined shipping protocols, including packaging standards, transport schedules, and contingency plans for delays, will be employed to maintain specimen integrity across inter-island and international routes.

Trial oversight within this hub-and-spoke model will include regular remote monitoring, training, and quality checks coordinated through UH Cancer Center research offices. Remote monitoring visits will review protocol adherence, data completeness, consent documentation, and reporting of adverse events at satellite sites, supplemented by targeted on-site visits as resources allow. Ongoing training delivered via virtual workshops, case discussions, and just-in-time refreshers will support satellite investigators, coordinators, and clinical staff in maintaining competence with protocol procedures and Good Clinical Practice standards.

By combining centralized regulatory and data functions with carefully staged activation of satellite and referring sites, Ka ʻUmeke Lama’s clinical research pillar seeks to extend access to innovative therapies while respecting the operational realities of small and remote health systems. Over time, lessons learned from early implementation will inform refinement of site readiness criteria, expansion of trial portfolios, and deeper integration of research into routine oncology care across Hawai’i and the USAPI.

### 4.3. Addressing Neighbor-Island and USAPI Health Disparities

The third pillar—addressing health disparities through clinical networks and community partnerships—integrates a statewide clinical oncology network with community-engaged prevention and early detection strategies. The initiative links community practices and hospitals with UH Cancer Center academic programs, leveraging telehealth to support subspecialty consultation, treatment planning, survivorship care, and clinical trial pre-screening. By design, this pillar focuses on extending the reach of subspecialty expertise to regions that historically lacked consistent access, while promoting adoption of shared care pathways that bridge community and academic settings.

Operational readiness is prioritized to ensure sustainability and consistency across sites, including investments in reliable connectivity, telehealth-friendly clinical workflows, and training for clinicians and staff in virtual care best practices. In parallel, Ka ʻUmeke Lama emphasizes evidence-based screening and early detection through active community engagement, including mobile and pop-up screening approaches where feasible and culturally responsive education developed with community leaders and organizations. Table 2 summarizes key regional challenges across Hawaiʻi and the USAPI and the corresponding Ka ʻUmeke Lama program responses, illustrating how structural barriers—geographic dispersion, workforce shortages, delayed diagnosis, and limited research infrastructure—are addressed through coordinated, system-level interventions.

Together, these three pillars form a coordinated, implementation-driven framework that promotes equity, expands access to innovation, and enhances cancer care and research capacity across Hawaiʻi and the USAPI. The subsequent evaluation framework makes these implementation dimensions explicit by linking them to concrete, trackable indicators.

## 5. Evaluation Framework and Proposed Metrics

Ka ʻUmeke Lama was designed from its inception with an explicit evaluation framework tied to implementation and accountability. Recognizing that complex system changes unfold over years, the program, albeit in its early implementation phase, integrates measurement into routine operations so that progress can be assessed objectively, disparities monitored transparently, and strategies adapted in response to real-world performance. The evaluation framework is structured around a set of indicators that map directly onto key implementation dimensions—reach, adoption, fidelity, and sustainability—as well as selected clinical and equity outcomes.

Process indicators primarily capture reach and adoption. For example, measures such as the number and geographic distribution of providers participating in Ka ʻUmeke Lama educational offerings, the number of sites engaged in tele-tumor boards, or the proportion of clinics using shared care pathways indicate the extent to which the program reaches intended targets and is taken up by clinicians and organizations. Similarly, metrics such as the number of sites with activated clinical trials, the proportion of patients from neighbor islands and the USAPI screened for trial eligibility, and participation in tele-oncology consultations reflect the reach of research and telehealth services, as well as the adoption of these modalities into practice.

Other process indicators are more tightly focused on fidelity. These include measures of adherence to agreed-upon care pathways (i.e., concordance with staging workup recommendations), use of standardized screening protocols, completion of mandated trial procedures at satellite sites, and consistency of documentation for navigation encounters and telehealth visits. Where feasible, audits of randomly sampled cases or automated EHR reports can be used to estimate fidelity to key pathway elements while allowing room for clinically appropriate local adaptation.

Indicators related to sustainability assess whether program components are maintained and institutionalized over time. Examples include the persistence of tele-oncology clinics and tumor boards beyond initial pilot phases, continued operation of fellowship rotations and regional teaching activities, retention rates of trained clinicians in neighbor-island and USAPI settings, and the stability or growth of dedicated staff positions for navigation, research coordination, and data management. Financial and organizational markers such as incorporation of Ka ʻUmeke Lama activities into health system budgets, long-term agreements with partner institutions, and sustained external funding are also viewed as signals of sustainability.

In parallel, the framework includes outcomes and equity indicators that reflect the initiative’s ultimate goals, while still recognizing the early-stage nature of implementation. Measures such as time from abnormal screening to diagnostic resolution, time to first oncology appointment, stage at diagnosis by geography and population group, and clinical trial enrollment stratified by island and race/ethnicity provide initial signals of whether greater reach, adoption, and fidelity are associated with improvements in care processes and equity. Patient-reported measures such as trust, satisfaction, and perceived coordination of care are planned for implementation in selected sites and, where collected longitudinally, will provide additional insight into patient-centered outcomes.

To support feasibility, each metric in Table 3 is accompanied by exemplar data sources and its primary implementation dimensions. For example,

- Reach: number of active partner sites, number of providers participating in education, number of tele-oncology encounters, proportion of neighbor-island/USAPI patients accessing subspecialty consultation. Data sources include workforce reports, program attendance logs, telehealth encounter data, and administrative claims.
- Adoption: proportion of clinics implementing standardized screening protocols, proportion of sites participating in tele-tumor boards, number of clinics with at least one activated trial. Data sources include site surveys, EHR configuration reports, and research office records.
- Fidelity: percentage of cases meeting pathway-defined staging or treatment elements, protocol deviation rates in satellite trial sites, completeness of navigation documentation. Data sources included EHR audits, trial monitoring reports, and quality dashboards.
- Sustainability: persistence of key activities over multiple years, retention of trained clinicians in targeted regions, incorporation of program costs into operating budgets, and continuity of external funding. Data sources include longitudinal workforce assessments, institutional budget documents, and grant/contract records.

Because data infrastructure and resources vary across Hawai’i and the USAPI, the framework explicitly anticipates phased implementation of metrics. In the initial phase, indicators that rely on existing administrative and clinical data systems will be prioritized, primarily within Hawai’i-based health systems and selected USAPI partners that meet predefined operational readiness criteria. The first phase of implementation will also include establishing a data collection process in order to generate baseline estimates for the target metrics. As data capabilities mature, additional metrics, particularly those requiring new patient-reported measures or detailed outcome stratification, will be gradually introduced.

By making explicit how each proposed metric maps onto reach, adoption, fidelity, and sustainability, the evaluation framework positions Ka ʻUmeke Lama not only as a descriptive model but also as a learning system. Routine, equity-focused monitoring of these indicators will inform iterative adjustments and support scholarly evaluation of the ecosystem as it evolves.

## 6. Discussion

A central strategic premise of Ka ʻUmeke Lama is that sustainable improvement in cancer outcomes cannot be achieved through isolated expansion of community-based clinical capacity alone. Simply increasing the number of community physicians or relying on financial incentives to recruit clinicians does not address the structural complexity of modern oncology care. High-quality cancer care increasingly depends on an integrated academic oncology ecosystem that deliberately balances community practice with research, education, and system-level evaluation.

Prior literature addressing cancer inequities in geographically isolated, rural, and underserved regions has consistently highlighted barriers related to access, workforce shortages, and limited participation in cancer research [15,31,32,33]. While outreach clinics, hub-and-spoke models, and tele-oncology have demonstrated benefits for access and patient satisfaction [20,34,35,36], these approaches are often implemented as discrete interventions rather than as components of an integrated, system-level academic framework. In Pacific Island and island-based settings, disparities in stage at diagnosis and outcomes are well documented, yet evaluable academic oncology ecosystem models remain scarce [22,37,38,39,40].

Although Ka ʻUmeke Lama is in its early implementation phase, it is distinguished by its intentional design as an implementation-focused academic oncology ecosystem rather than a site-specific or disease-specific program [41]. Its strength lies in aligning academic oncology expertise with community-based care delivery across Hawaiʻi and the USAPI through standardized care pathways, longitudinal workforce training, and coordinated research operations [42,43]. Equally important, the initiative explicitly incorporates non-physician professionals—including oncology nurses, advanced practice providers, pharmacists, social workers, patient navigators, and allied health staff—recognizing that multidisciplinary teams are foundational to delivering guideline-concordant, patient-centered cancer care. In addition, data scientists, informaticians, and analytics specialists are essential members of the oncology ecosystem, supporting tumor board decision-making through data integration, enabling pre-screening for clinical trials, and advancing equity-stratified evaluation within a learning health system framework.

The program also embeds measurement and accountability from its inception. By linking process indicators to outcomes and equity metrics stratified by geography and population group, Ka ʻUmeke Lama enables continuous learning, adaptive management, and scholarly evaluation [41,42]. This approach moves beyond aspirational commitments to equity by providing a transparent framework to assess whether implementation efforts translate into meaningful improvements for populations experiencing the greatest disparities. The integration of data science expertise into multidisciplinary oncology teams is critical to improving trial access, care coordination, and equity monitoring, particularly in resource-constrained and geographically dispersed settings.

Several limitations warrant consideration. Ka ʻUmeke Lama is described as a conceptual and early-stage implementation model rather than a mature, fully evaluated initiative. As a result, all expectations regarding improved cancer care or reduced disparities should be interpreted as aspirational and contingent upon future evaluation rather than as established effects. Outcomes such as changes in stage at diagnosis, survival, and long-term workforce retention will require multi-year follow-up and cannot be fully assessed in early phases [22,37]. Implementation is also expected to vary substantially across sites due to heterogeneous infrastructure, underscoring the importance of ongoing evaluation and flexibility [33,40]. These differences will inevitably influence the pace, scope, and fidelity of implementation, limiting the generalizability of specific strategies and requiring context-specific adaptation rather than simple replication. In addition, hub-and-spoke and tele-mentoring models may introduce risks of workforce strain and burnout, particularly for specialists at hub sites who provide distributed support across multiple regions. Similarly, the proposed evaluation framework is ambitious and may not be fully realizable in all settings. Several metrics, particularly those that rely on detailed, equity-stratified clinical outcomes or patient-reported measures of trust and satisfaction, may be incompletely captured or subject to substantial measurement error in jurisdictions with limited data systems or inconsistent documentation. Furthermore, variability in digital infrastructure across sites may not only limit implementation fidelity but also introduce new forms of inequity, particularly in telehealth access, data capture, and clinical trial participation. Finally, the long-term financial sustainability and workforce implications of Ka ʻUmeke Lama extend beyond the control of the program’s academic sponsors. While the model is intentionally aligned with existing initiatives and seeks to leverage current resources, its durability will ultimately be influenced by policy and funding decisions at institutional, state, federal, and regional levels.

Despite its grounding in the specific geographic and cultural context of Hawaiʻi and the Pacific, the core principles underlying Ka ʻUmeke Lama have broader relevance. Regions characterized by geographic dispersion, limited specialty workforce, and culturally diverse populations may benefit from similar ecosystem-based approaches that integrate workforce pipelines, telehealth-enabled clinical networks, standardized research infrastructure, and embedded evaluation [15,21,27,28,42,43]. By documenting implementation processes and outcomes, Ka ʻUmeke Lama can contribute to a more generalizable evidence base on how to design and sustain academic oncology ecosystems in resource-constrained settings.

### Strategic Implications

Ka ʻUmeke Lama highlights an important lesson for health systems and policymakers: physician recruitment alone is insufficient to resolve oncology access and equity gaps. Sustainable progress requires deliberate development of academic oncology infrastructure in parallel with community practice, including investment in multidisciplinary teams and strong partnerships with schools of nursing, social work, pharmacy, and other health professions. At the same time, many underserved regions lack the resources to build such systems independently, necessitating shared academic–community models rather than isolated local solutions.

The initiative also directly confronts a long-standing vicious cycle in Hawaiʻi and the Pacific, in which talented trainees are educated locally but leave the region without returning. As national organizations such as the American Society of Clinical Oncology (ASCO) increasingly define education and clinical trial participation as essential components of standard oncology care, failure to create environments that integrate academic opportunity with community impact risks accelerating brain drain and widening disparities. By aligning education, research, and clinical care within a unified regional framework, Ka ʻUmeke Lama offers a pragmatic strategy to retain talent, strengthen local capacity, and advance equitable cancer care.

Looking forward, the strategic implications extend beyond oncology. The principles of integrated academic–community ecosystems, telehealth-enabled networks, workforce pipelines, and embedded evaluation may be applicable to other complex, multidisciplinary areas of care such as cardiology, neurology, and maternal–fetal medicine. For Hawai’i and the USAPI, Ka ʻUmeke Lama thus serves not only as a cancer-specific initiative but also as a model for how geographically isolated regions can leverage academic partnerships to transform care delivery more broadly.

## 7. Conclusions

Ka ʻUmeke Lama represents a structured, implementation-oriented approach designed to build an academic oncology ecosystem that addresses cancer inequities across Hawaiʻi and the U.S.-Affiliated Pacific Islands. By integrating academic oncology with community practice, multidisciplinary workforce development, clinical research infrastructure, and embedded evaluation, the initiative moves beyond isolated interventions toward sustainable system-level transformation. The program’s three pillars—education and workforce development, clinical research and access to innovation, and clinical networks with community partnerships—are designed to operate synergistically to expand access, enhance quality, and center equity in cancer care across a geographically dispersed, culturally diverse region.

This framework provides a pragmatic and transferable model for regions facing geographic dispersion, workforce shortages, and persistent inequities in cancer care. As implementation proceeds, ongoing measurement and community engagement will be essential to ensure that Ka ʻUmeke Lama remains responsive to evolving needs and that its benefits are equitably distributed across counties, islands, and population groups. In doing so, the initiative seeks not only to transform cancer care in Hawai’i and the Pacific but also to contribute to a broader reimagining of how academic oncology can partner with communities to achieve equity and excellence in cancer control.

## Figures and Tables

**Table 1:** Health disparity tiers—UH Cancer Center catchment area.

Relative Disparity Level	Geographic Area	Key Characteristics	Primary Barriers	Strategic Focus for UH Cancer Center
Lowest	East Oʻahu	Highest density of tertiary care services; proximity to academic medical centers; higher insurance coverage	Time constraints, system navigation complexity	Quality optimization, clinical trial enrollment, precision oncology, survivorship
Moderate	Non-East Oʻahu	Geographic access within Oʻahu but farther from specialty centers; more socioeconomic diversity	Transportation, delayed diagnosis, care coordination gaps	Care coordination, navigation, community-based screening, decentralized trials
High	Neighbor Islands (Maui, Hawaiʻi Island, Kauaʻi, Molokaʻi, Lānaʻi)	Limited oncology workforce; reliance on inter-island travel; smaller health systems	Travel burden, delayed treatment, limited trial access	Tele-oncology, hub-and-spoke models, mobile screening, regional trial activation
Highest	U.S.-Affiliated Pacific Islands (USAPI)	Resource-limited settings; small populations spread across vast geography; cultural and language diversity	Severe access limitations, infrastructure gaps, workforce shortages	Capacity building, regional partnerships, prevention and early detection, global oncology leadership

**Table 2:** Key regional challenges and Ka ʻUmeke Lama program responses.

Regional Challenge	Ka ʻUmeke Lama Program Response
Geographic dispersion across islands and remote atolls	Network-based care pathways, expanded telehealth consultation, coordinated referral systems, and shared clinical protocols to ensure continuity of care
Shortages in physicians and oncology-specialized workforce	Oncology-focused training pathways, multidisciplinary team development, and targeted recruitment and retention strategies
Limited screening access and delayed diagnosis in underserved populations	Community-engaged outreach initiatives, culturally responsive screening education, and patient navigation support
Underdeveloped clinical trials and research infrastructure outside Honolulu	Standardized clinical trial operations, remote trial pre-screening, and partnership-based trial activation across sites
Population aging and increasing complexity of cancer care	Geriatric-informed care models, supportive and palliative care integration, and ongoing workforce upskilling

**Table 3:** Proposed evaluation metrics for Ka ʻUmeke Lama.

Domain	Process Indicators	Outcomes/Equity Indicators
Workforce and training	Trainees/faculty in oncology pathways; multidisciplinary training sessions; tele-mentoring sessions	Oncology FTEs by county/island; retention; time-to-appointment
Access and care coordination	Telehealth consult volume; referral completion; navigation encounters	Time from abnormal screening to diagnosis; diagnosis to treatment start; travel burden
Screening and early detection	Outreach events; screening sites activated; navigator outreach	Screening uptake; stage at diagnosis by geography and population group
Clinical trials and research	Trials activated; participating sites; pre-screening volume	Trial accrual by geography and population group; reasons for non-enrollment
Patient experience and quality	PRO collection; interpreter utilization; education materials deployed	Patient-reported access/trust/satisfaction; treatment adherence/continuity

## Data Availability

No new data were created or analyzed in this study. Data sharing is not applicable to this article.
